# Systemic dissemination of chronic necrotizing pulmonary aspergillosis in an elderly woman without comorbidity: a case report

**DOI:** 10.1186/1752-1947-6-270

**Published:** 2012-08-31

**Authors:** Kotaro Tokui, Yukio Kawagishi, Minehiko Inomata, Chihiro Taka, Seisuke Okazawa, Toru Yamada, Toshiro Miwa, Ryuji Hayashi, Shoko Matsui, Yasuo Takano, Kazuyuki Tobe

**Affiliations:** 1 Department of Internal Medicine, University of Toyama, 2630 Sugitani, Toyama, 930-0194, Japan; 2 Department of Diagnostic Pathology, University of Toyama, 2630 Sugitani, Toyama, 930-0194, Japan

## Abstract

**Introduction:**

Chronic necrotizing pulmonary aspergillosis usually occurs in mildly immune-compromised hosts or those with underlying pulmonary disease. The radiographic pattern of chronic necrotizing pulmonary aspergillosis is typically a progressive upper lobe cavitary infiltrate with pleural thickening. We report here an atypical case of chronic necrotizing pulmonary aspergillosis mimicking lung cancer, which developed into a disseminated fatal disease in an older woman with no comorbidity.

**Case presentation:**

An 80-year-old Japanese woman was referred to our hospital for a chest roentgenogram abnormality. Repeated fiber-optic bronchoscopy could not confirm any definite diagnosis, and she refused further examinations. Considering the roentgenogram findings and her age, she was followed-up as a suspected case of lung cancer without any treatment. Then, 10 months later, she complained of visual disturbance and was admitted to our department of ophthalmology. She was diagnosed as having endophthalmitis. After treatment with corticosteroids for 20 days, she developed acute encephalitis and died four weeks later. Autopsy revealed dissemination of *Aspergillus* hyphae throughout her body, including her brain.

**Conclusions:**

In older patients, even if they do not have any comorbidity, chronic necrotizing pulmonary aspergillosis should be added to the differential diagnosis of solitary pulmonary lesions in a chest roentgenogram.

## Introduction

*Aspergillus* causes a variety of clinical syndromes in the lung, such as aspergilloma, chronic necrotizing pulmonary aspergillosis (CNPA), invasive pulmonary aspergillosis and allergic bronchopulmonary aspergillosis [1]. Aspergilloma occurs in patients with lung cavities, CNPA occurs in those who are mildly immunocompromised and invasive pulmonary aspergillosis is seen in patients who are immunocompromised, such as those with leukemia. We report a case of an older woman with a tumor-like shadow in her chest roentgenogram, which developed into disseminated invasive aspergillosis after 10 months of follow-up.

## Case presentation

**Figure 1:**
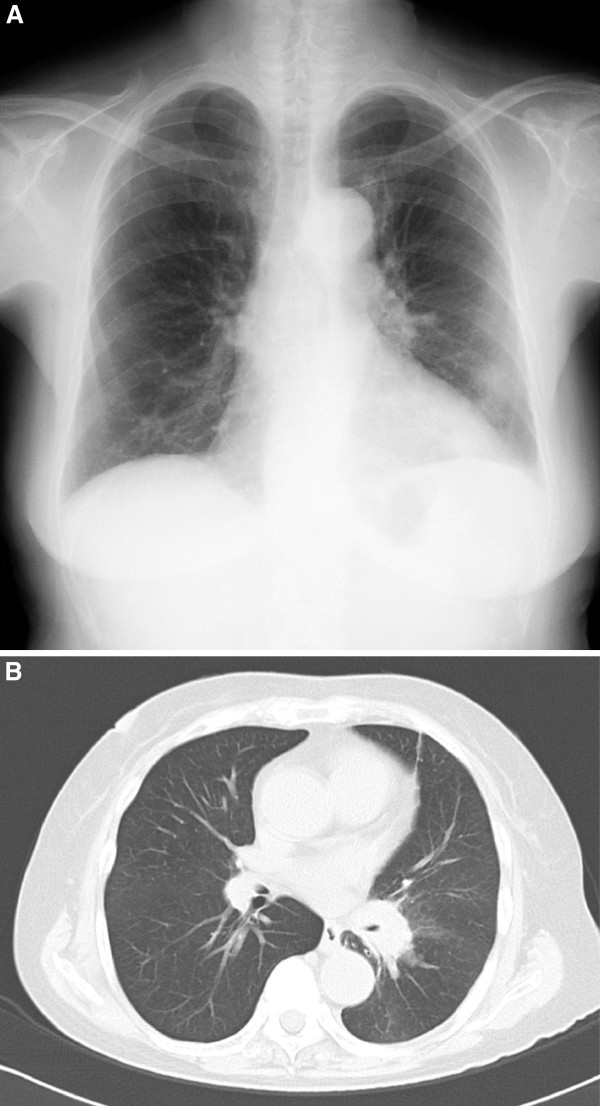
**Chest imaging findings. (A)** Posterior anterior view of the chest roentgenogram showing the left hilar and para-aortic tumor-like shadows. **(B)** Chest computed tomography (CT) imaging showing the tumor-like shadow surrounding the left lower bronchus.

**Figure 2:**
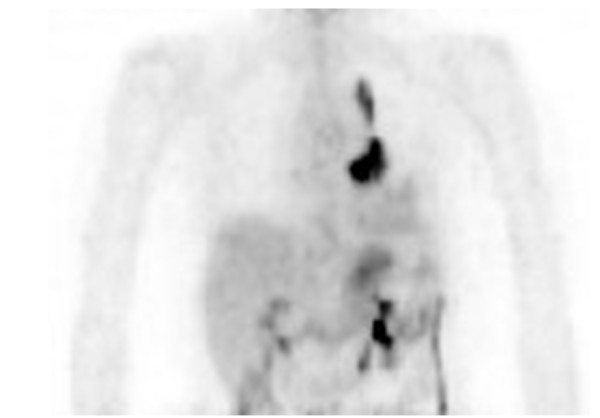
Positron emission tomography (PET) imaging showing the uptake of FDG at the lower and upper lesions in the left lung.

An 80-year-old Japanese woman, without any relevant medical history, was referred to our hospital for a chest roentgenogram abnormality. Her chest roentgenogram and computed tomography (CT) results revealed tumor-like shadows, one of which was 35mm in diameter with a clear margin (Figure 1). It did not have a halo sign. F-fluorodeoxyglucose (FDG) positron emission tomography showed strong accumulation of glucose in tumor-like lesions (standardized uptake values (SUVs) of 8.53 and 21.1; Figure 2). She underwent two fiber-optic bronchoscopy examinations. However, the endobronchial biopsy specimen could not confirm any definite diagnosis. Owing to her age, further aggressive examination was not performed and she was followed-up as having suspected lung cancer. Her lung lesions gradually developed, which was compatible with lung cancer progression. Her general condition was well and there was no sign of infectious disease. Then, 10 months after her first visit, she complained of visual disturbance and eye pain, and was admitted to the department of ophthalmology of our hospital. Her clinical findings revealed endophthalmitis, but its etiology was unknown. She was treated with corticosteroids urgently for her rapidly progressing eye symptoms. She was administered fluconazole because systemic examination revealed esophageal candidiasis. At 20 days after admission, she developed acute encephalitis and was transferred to our department. Her serum level of β-d glucan was high (1184pg/mL) and a test result for *Aspergillus* antigen was positive. She was diagnosed as having invasive aspergillosis at this time and treated with micafungin followed by voriconazole. She died four weeks after the onset of encephalitis.

**Figure 3:**
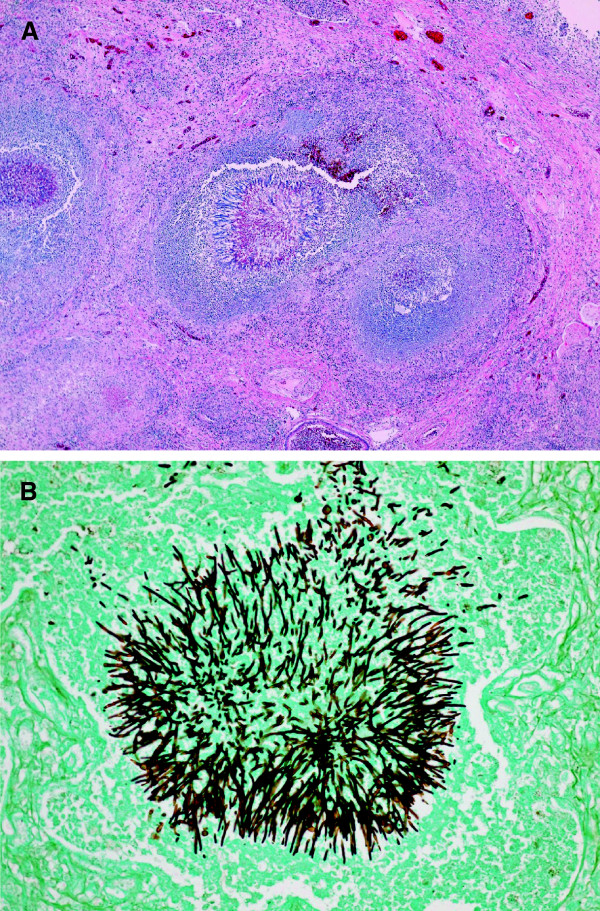
**Histopathological findings. (A)** Collection of fungal hyphae surrounded by inflammatory cells and granulation tissue in the lung lesion; hematoxylin and eosin stain (×40). **(B)** Fungal hyphae stained by Groccot’s stain (×200).

An autopsy was performed after her death with her relatives’ consent. The primary lesion of the lung consisted of fungus hyphae characteristic of *Aspergillus*. Inflammatory cells such as lymphocytes, plasma cells and eosinophils as well as granulation tissue surrounded the fungus lesion (Figure 3). A histologic examination of other organs including the cerebrum revealed that *Aspergillus* hyphae had disseminated throughout her body. The pathological diagnosis was disseminated invasive aspergillosis.

## Discussion

The disease type associated with *Aspergillus* infection depends on the host’s immunological status. Invasive pulmonary aspergillosis usually occurs in immunocompromised hosts, such as patients with leukemia, and is rapidly progressive [2]. The predisposing factors for CNPA development are mildly immunocompromised status or underlying lung diseases, such as chronic obstructive pulmonary disorder (COPD), inactive tuberculosis and pneumoconiosis [3,4]. CNPA is usually a chronic, indolent and localized form of *Aspergillus* infection [5]. Our patient’s case was compatible with this category, but was not typical in two regards. First, our patient did not have any underlying lung disease. Second, she developed disseminated fatal disease in the course of CNPA. We speculate that her age contributed to the disease. Cornet *et al*. reported two cases of fulminant invasive pulmonary aspergillosis in immunocompetent but older patients [6]. Two patients received short-term systemic corticosteroid therapy and their invasive aspergillosis progressed rapidly. The authors speculated that old age may have been a predisposing factor for the onset and rapidly fatal outcome of invasive aspergillosis. It should be highlighted that very old age might be a predisposing factor of CNPA. Two groups in Korea reported pulmonary aspergillosis in immunocompetent hosts without underlying lung disease [7,8]. According to these authors, radiographic findings presented as a nodule or mass that could not be differentiated from malignancy on CT scan. Similarly, the radiographic findings in our patient’s case mimicked lung cancer. The image of another case of invasive aspergillosis was also reported to mimic lung cancer [9]. Because lung cancer is far more prevalent than aspergillosis, the latter might be easily misdiagnosed as lung cancer. Because anti-fungal treatments have improved recently [10], an appropriate diagnosis and early treatment of CNPA are particularly important for a better outcome.

## Conclusions

A tumor-like shadow in a chest roentgenogram can be caused by CNPA in older people with no comorbidity. It should be borne in mind that very old age might itself be a predisposing factor for CNPA. Observation without appropriate treatment and even short-term corticosteroid therapy might lead to acute fatal disseminated aspergillosis. It is important to undertake careful examination and observation of undiagnosed pulmonary nodular or mass lesions because CNPA can be hidden in older patients.

## Consent

Written informed consent was obtained from the patient’s next of kin for publication of this case report and any accompanying images. A copy of the written consent is available for review by the Editor-in-Chief of this journal.

## Competing interests

The authors declare that they have no competing interests.

## Authors’ contributions

KT and YK wrote the manuscript. MI, CT, SO, TY, TM and SM helped treat our patient. YT contributed to the histological diagnosis. RH was a major contributor in writing the manuscript. KT managed the consent and writing of the manuscript. All authors have read and approved the final version of the manuscript.

## References

[B1] Soubani AO Chandrasekar PH The clinical spectrum of pulmonary aspergillosis Chest 2011 121 1988 1999 10.1378/chest.121.6.1988 12065367

[B2] Gottfredsson M Steingrimsdottir H Disseminated invasive aspergillosis in a patient with acute leukemia Acta Biomed 2006 77 S10 S13 16918060

[B3] Gefter WB Weingrad TR Epstein DM Ochs RH Miller WT “Semi-invasive” pulmonary aspergillosis Radiology 1981 140 313 321 7255704 10.1148/radiology.140.2.7255704

[B4] Binder RE Faling LJ Pugatch RD Mahasaen C Snider GL Chronic necrotizing pulmonary aspergillosis: a discrete clinical entity Medicine 1982 61 109 124 10.1097/00005792-198203000-00005 7038373

[B5] Kondo T Nishiya K Kobayashi I Fukui T Tazaki G Ishii H Yanagimachi N A case of pulmonary semi-invasive aspergillosis developing fatal acute exacerbation Tokai J Exp Clin Med 2006 31 91 95 21302231

[B6] Cornet M Mallat H Somme D Guerot E Kac G Mainardi JL Fores P Gutmann L Lavarde V Fulminant invasive pulmonary aspergillosis in immunocompetent patients - a two-case report Clin Microbiol Infect 2003 9 1224 1227 10.1111/j.1469-0691.2003.00792.x 14686988

[B7] Kang EY Kim DH Woo OH Choi JA Oh YW Kim CH Pulmonary aspergillosis in immunocompetent hosts without underlying lesions of the lung Am J Radiol 2002 178 1395 1399 10.2214/ajr.178.6.1781395 12034604

[B8] Yoon SH Park CM Goo JM Lee HJ Pulmonary aspergillosis in immunocompetent patients without air-meniscus sign and underlying lung disease: CT findings and histopathologic features Acta Radiol 2011 52 756 761 10.1258/ar.2011.100481 21596796

[B9] Wilkinson MD Fulham MJ Mccaughan BC Constable CJ Invasive aspergillosis mimicking stage IIIA non-small-cell lung cancer on FDG positron emission tomography Clin Nucl Med 2003 28 234 235 12592137 10.1097/01.RLU.0000053535.03453.D9

[B10] Limper AH Knox KS Sarosi GA Ampel NM Bennett JE Catanzaro A Davies SF Dismukes WE Hage CA Marr KA Mody CH Perfect JR Stevens DA An official American thoracic society statement: treatment of fungal infections in adult pulmonary and critical care patients Am J Respir Crit Care Med 2011 183 96 128 10.1164/rccm.2008-740ST 21193785

